# A dataset for energy demand and supply modelling in Sierra Leone

**DOI:** 10.1016/j.dib.2024.110715

**Published:** 2024-07-08

**Authors:** Fynn Kiley, David Caulker, William Collier, Neve Fields, William Blyth, Jairo Quirós-Tortós, Mark Howells

**Affiliations:** a STEER Centre, Department of Geography, Loughborough University, Loughborough, LE11 3TU, United Kingdom; b University of Sierra Leone, Fourah Bay College, Barham Road, Southern Central, Freetown, Sierra Leone; c Ministry of Energy, 36 Siaka Stevens St, Freetown, Sierra Leone; d Foreign Commonwealth and Development Office, King Charles St, London SW1A 2AH, United Kingdom; e Centre for Environmental Policy, Imperial College London, London, SW7 2BX, United Kingdom

**Keywords:** Electricity, Hydropower, Grid, Data, MAED, OSeMOSYS, Least cost

## Abstract

Energy systems modelling plays a pivotal role in understanding and optimizing complex energy systems. By integrating various factors such as energy demand, supply, infrastructure, and environmental considerations, energy systems modelling provides valuable insights for policymakers, industry stakeholders, and researchers. This can be key to informing stakeholder and policy decisions and facilitate the mobilisation of capital and market development to support the development of the energy sector. This article presents the data, assumptions, and related calculations used for the development of a national scale power system model for Sierra Leone. The focus of the model was a techno-economic analysis of specific hydropower expansion plans. Where possible, this data has been collected from publicly available sources such as scientific literature, feasibility studies, international databases, data from existing modelling efforts in Sierra Leone, and local stakeholders. The collection of the data, development of the model, and creation of scenarios was done in collaboration with the Ministry of Energy in Sierra Leone and verified by a broad range of stakeholders.

The following paper outlines the key data presented in the full database which can be accessed through the link found in the Specifications Table. The link also contains the Reference Energy System (RES) used in this study.

Specifications Table

**Table:** 

Subject	Renewable Energy, Sustainability, and the Environment
Specific subject area	Energy system modelling
Type of data	Table, Graph. Raw, Analysed, Processed.
Data collection	This data has been collected from publicly available sources such as scientific literature, feasibility studies, international databases, data from existing modelling efforts in Sierra Leone, and local stakeholders. Data was collected from relevant State-Owned Enterprises (SOEs) with the help of the Ministry of Energy in Sierra Leone, who helped to define the scope of the energy system model and requested data from relevant power sector stakeholders. Scenarios were also developed in collaboration with the Ministry of Energy in Sierra Leone to ensure that potential power projects such as plans to further develop the Bumbuna sites aligned with projects currently considered by the Government of Sierra Leone in strategies to further develop their power sector.
Data source location	Collection methodology, raw data sources, and assumptions are listed in Section 3 of this article. Data was collected primarily through government and utility databases and collection from secondary sources for historical datapoints. Values of parameters for future years were derived from trends seen in secondary sources. The model period covered was 2018–2050.
Data accessibility	Repository name: Zenodo. Data identification number: 10.5281/zenodo.10974793 Direct URL to data: https://doi.org/10.5281/zenodo.10974793

Description of parameters and acronyms

**Table:** 

**Parameters**	**Description**

Final Energy Demand	Final energy used by consumers (Residential, Service, Industrial)
Capital Costs	One-off capital investment cost for the construction of each technology per unit of capacity.
Operational Life	Period between completed construction and decommissioning of a technology in years.
Fixed Cost	The fixed annual cost associated with the operation and maintenance of each technology per unit of capacity
Variable Cost	The cost of fuel associated with the operation of a power plant per unit of activity
Emissions Factors	An approximation for the quantity of pollutant (CO_2_) released relative to the use of one unit of fuel.
Efficiencies	The ratios between energy input from each fuel source, and the energy output from each technology.
Capacity Factors	The ratio of energy produced by a technology as a factor of potential energy production assuming continuous operation of each technology throughout the same period.
Residual Capacities	Existing or committed technology capacities in Sierra Leone's power sector.
Annual Potentials and Reserves	The availability of renewable and non-renewable resource potentials in Sierra Leone, measured in units of energy.
**Acronym**	**Description**

BAU	Business as Usual
CCGT	Combined Cycle Gas Turbine
CLSG	Côte d'Ivoire-Liberia-Sierra Leone-Guinea Transmission Line
EDSA	Electricity Distribution and Supply Authority
EGTC	The Electrical Generation and Transmission Company
GDP	Gross Domestic Product
GWh	Gigawatt Hours
HFO	Heavy Fuel Oil
IAEA	International Atomic Energy Agency
IEA	International Energy Agency
IRENA	International Renewable Energy Agency
IRP	Integrated Resources Planning
LFO	Light Fuel Oil
MAED	Model for Analysis of Energy Demand
MoE SL	Ministry of Energy Sierra Leone
OSeMOSYS	The Open-Source Energy Modelling System
PJ	Petajoules
PV	Photovoltaic
RDM	Robust Decision Making
SCGT	Simple Cycle Gas Turbine
SLEWRC	Sierra Leone Electricity and Water Regulatory Commission
TEMBA	The Electricity Model Base for Africa

## 1 Value of the Data

• The aim of this data is to enable the continuation of modelling and in-country capacity building within Sierra Leone and the Ministry of Energy.
• The data collection approach used in constructing this database helps to bridge the gaps in current literature regarding demand projections and development of power generation capacity in Sierra Leone, helping address issues around ensuring year-round energy supply through better informed policy making and project selection.
• This can also inform the national needs for investment in key technologies and projects within Sierra Leone, as exemplified by the ‘Data-to-Deal’ framework [1].
• Robust Decision Making (RDM) allows for the stress-testing of key assumptions regarding projected parameters, helping to validate the outcomes of OSeMOSYS modelling.
• This data can be useful for teaching, consultation, and policy development in Sierra Leone, to facilitate open and accessible evidence-driven policy development and scenario construction, with the flexibility of OSeMOSYS allowing for the addition of further end uses and sectors such as cooking and transport in the future.
• Open-source data allows for more transparent and accessible analysis and policy assessment.

## 2 Background

The energy sector in Sierra Leone is currently in a period of crisis with inadequate generation capacity, inefficient transmission and distribution infrastructure, low electrification rates in rural and urban populations, and frequent power outages [2]. Furthermore, during the dry season the country relies on the Karpowership Heavy Fuel Oil (HFO) power barge to provide the majority of generating capacity. Due to unpaid arrears, the output of the barge is often reduced to provide power to essential services only, turning off the lights for households, non-essential services, and industry [3].

The aim of this open dataset is to encourage increased transparency and accessibility for the ongoing collaborative development of energy sector modelling within Sierra Leone, both within Ministries and research and modelling groups, and to promote more rigorous and well-founded policy development and management. Additionally, it is designed to establish a foundation from which to develop further in-country modelling capacity. While previous modelling has been conducted on Sierra Leone's power sector by the Millennium Challenge Corporation and Adam Smith International, the input data and assumptions and the results have not been made publicly available. To the best knowledge of the authors, a comprehensive dataset detailing Sierra Leone-specific techno-economic and social data for energy modelling, as well as a detailed account of the assumptions made and why, as presented in this article, does not currently exist in the public sphere.

## 3 Data Description

The data provided in this report can be used to replicate the Open-Source Modelling System (OSeMOSYS) and associated Data-to-Deal framework which also includes the Model for Analysis of Energy Demand (MAED) for Sierra Leone. OSeMOSYS is an open source, bottom-up energy system cost optimisation tool that meets all defined energy demands (e.g. on grid electricity) and additional constraints (e.g. resource potentials, emission constraints, etc.) in its solutions to any scenarios under investigation [4]. MAED is a simple and easy to use energy demand forecasting model developed by the International Atomic Energy Agency that uses a bottom-up simulation methodology [5]. Following the Data-to-Deal analytical workflow [1], the final energy demand outputs from MAED were used as inputs for the OSeMOSYS model to define the demand [6]. While MAED outputs include final and useful energy demands, final energy demand was used in this instance to allow OSeMOSYS to optimise the required generation mix.

This report outlines key inputs from this modelling, including a RES to indicate how the model was constructed, and what assumptions were made to facilitate this. The data collected for use in this model was primarily sourced from publicly available resources and was collected and processed in accordance with the procedure outlined in the OSeMOSYS data collection methodology [7]. The full dataset and Reference Energy System (RES) can be accessed through the link found in the Specifications Table.

### 3.1 Demand projections

To model demand in Sierra Leone's power sector, previous modelling conducted for the country's Integrated Resource Plan (IRP) was used to inform a subsequent MAED model. To undertake this, baseline historic on-grid generation data was collected from the Electricity Distribution and Supply Authority (EDSA) with collaboration with the Electricity Generation and Transmission Company (EGTC). This was adjusted by transmission and distribution efficiencies found in Table 20, to represent the final energy demand between 2018 and 2023 found in Table 1. From the IRP data, compound annual growth rates (CAGR) and sectoral share was calculated for each scenario found in Table 2, and applied to the historic data to produce demand projections between 2023 and 2050 summarised in Table 3. The complete dataset for the IRP demand projections modelled in Sierra Leone can be found in the dataset found in the repository in the Excel File “*Sierra Leone Model Data*” in the sheet labelled “*1. IRP Demand*” with results found in “*4. Demand Results*”.

**Table 1:** A list of all the tables containing summaries of the data presented in the paper, including a description of their contents.

Item	Description of Contents
Table 2.	On grid electricity demand in Sierra Leone from 2018 to 2023.
Table 3.	Electricity demand growth rates and the share of electricity demand for each sector under the Base, High and Low demand scenarios found in Sierra Leone's Integrated Resource Plan (IRP).
Table 4.	A summary of historic and projected demographic parameters from 2018 to 2030 including population, population growth rate, urban-rural population split, people per urban and rural household, and potential and participating labour force.
Table 5.	A summary of historic and projected Gross Domestic Product (GDP) parameters from 2018 to 2028 including total GDP, annual GDP growth rates, and sectoral shares of GDP.
Table 6.	Electricity consumption in Agriculture, Mining, Services, and urban and rural households from 2018 to 2023.
Table 7.	Urban and rural household Electrification rates from 2018 to 2050.
Table 8.	Historic and predicted sectoral energy intensities per annum from 2018 to 2050.
Table 9.	Base, High, and Low GDP growth rate scenarios from 2023 to 2050.
Table 10.	Base, High, and Low population growth rate scenarios from 2023 to 2050.
Table 11.	Projected generation technology capital costs in $/kW from 2023 to 2050.
Table 12.	Projected transmission and distribution technology capital costs in USD/kW from 2023 to 2050.
Table 13.	Estimated electricity generation technology operational life.
Table 14.	Estimated transmission and distribution technology operational life.
Table 15.	Projected electricity generation technology fixed costs from 2023 to 2050 in USD/kW/year.
Table 16.	Projected transmission and distribution technology fixed costs from 2023 to 2050.
Table 17.	Fuel cost projections in USD/GJ for imported and domestically extracted fuels from 2024 to 2050.
Table 18.	Emissions factors for the fuels used in the modelling in kg of CO2e per GJ.
Table 19.	Estimated electricity generation technology average efficiencies in%.
Table 20.	Historic and projected efficiencies for transmission and distribution infrastructure from 2023 to 2050.
Table 21.	Estimated electricity generation technology average capacity factors in%.
Table 22.	Estimated transmission and distribution technology average capacity factors in Sierra Leone.
Table 23.	Electricity generation technology residual capacities and capacities of committed plants, grouped by technology when relevant, from 2021 to 2050 in MW.
Table 24.	Residual capacities for transmission and distribution technologies from 2021 to 2050 in MW.
Table 25.	Fossil fuel and renewable resource potentials for the power sector in Sierra Leone.
Table 26.	Existing and proposed Bumbuna reservoir energy potentials.
Table 27.	On and off grid historic power generation in Sierra Leone from 2018 to 2050 in MWh.
Table 28.	Variable input adjustments and upper and lower bounds for Robust Decision-Making (RDM) modelling.
Table 29.	List of sources and assumptions for the Sierra Leone population demographic parameters (2018–2030)
Table 30.	List of sources and assumptions for the Sierra Leone GDP parameters (2018–2028)
Table 31.	List of sources and assumptions for electricity consumption per sector (2018–2023)
Table 32.	List of sources and assumptions for electrification rates (2018–2050)
Table 33.	List of sources and assumptions for GDP growth rate scenarios for Sierra Leone (2023–2050)
Table 34.	List of sources and assumptions for population growth rate scenarios for Sierra Leone
Table 35.	List of sources and assumptions for technology capital costs
Table 36.	List of sources and assumptions for technology lifetimes
Table 37.	List of sources and assumptions for technology fixed costs
Table 38.	List of sources and assumptions for technology variable costs
Table 39.	List of sources and assumptions for technology efficiencies
Table 40.	List of sources and assumptions for average technology capacity factors
Table 41.	List of sources and assumptions for annual potential and reserves in Sierra Leone.
Table 42.	List of sources and assumptions for historic generation data in Sierra Leone (2018–2023)
Figure 1.	Graphs showing historic and projected demand growth based on IRP data and EDSA historic generation data from Table 1, Table 2. including sectoral breakdown between Household, Service and Industrial sectors.
Figure 2.	MAED projected final energy demand under base, high and low scenarios using data from Table 4, Table 5, Table 6, Table 7, Table 8, Table 9, Table 10.

**Table 2:** On grid electricity demand in Sierra Leone from 2018 to 2023.

	Final Annual Electricity Demand (GWh)					
	2018	2019	2020	2021	2022	2023
Electricity Demand	182.083*	284.188*	250.424*	262.163*	324.909*	328.586*

⁎ These values were derived from real-world historic data provided by EDSA.

**Table 3:** Electricity demand growth rates and the share of electricity demand for each sector under the Base, High and Low demand scenarios found in Sierra Leone's Integrated Resource Plan (IRP).

Scenario	Growth Rates (%/yr) and Share of Demand (% in 2019)						
	Total CAGR (%)	Household CAGR (%)	Commercial CAGR (%)	Industrial CAGR (%)	Household Share (%)	Commercial Share (%)	Industry Share (%)
Base	7.21 %	9.21 %	4.09 %	9.92 %	31.22 %	53.38 %	15.40 %
High	9.59 %	11.62 %	6.39 %	12.36 %	31.22 %	53.38 %	15.40 %
Low	6.19 %	8.16 %	3.09 %	8.87 %	31.22 %	53.38 %	15.40 %

The recalibrated IRP demand projections were used to inform the development of the MAED model. MAED is a scenario-based tool developed by the International Atomic Energy Agency (IAEA) that uses current econometric data to generate projected estimates of annual energy demand on a national scale. While not used in the example energy-system analysis, Table 4, Table 5, Table 6, Table 7, Table 8, Table 9, Table 10 outline the methodology for the collection of data required for modelling demand in Sierra Leone's power sector in line with the “*Data-to-Deal*” pipeline [1]. This can be expanded to include sectors such as transport if included in the OSeMOSYS modelling; however, the data currently outlined in the following tables is limited to Sierra Leone's power sector. The complete dataset for this section can be found in the data repository in the Excel File “*Sierra Leone Model Data*” under the sheet labelled “*2. MAED Demand*”.

**Table 4:** A summary of historic and projected demographic parameters from 2018 to 2030 including population, population growth rate, urban-rural population split, people per urban and rural household, and potential and participating labour force.

Variable	Units	Summary of Demographic Parameters								
		2018	2019	2020	2021	2022	2023	2024	2025	2030
Population	Million	7.861								
Population Growth Rate	%	–	2.13	2.09	2.06	2.06	2.06	2.06	2.06	2.07
Urban Population	%	42	42	43	43	44	44	45	45	50
Person/Urban Household	Capita	5	5	5	5	5	5	5	5	5
Person/Rural Household	Capita	6	6	6	6	6	6	6	6	6
Potential Labour Force	%	72	72	72	72	72	72	72	72	72
Participating Labour Force	%	54	54	54	54	54	54	54	54	54

**Table 5:** A summary of historic and projected Gross Domestic Product (GDP) parameters from 2018 to 2028 including total GDP, annual GDP growth rates, and sectoral shares of GDP.

Variable / Sector	Units	Summary of GDP Parameters								
		2018	2019	2020	2021	2022	2023	2024	2025	2028
GDP (US$ Billion)	US$ Millions	4.09								
GDP Growth Rate	%		5.25	−2	4.1	3.98	2.75	4.74	5.19	4.6
Sectoral Shares of GDP										
Industry	%	63.3	63.3	63.3	63.3	63.3	63.3	63.3	63.3	63.3
Agriculture	%	57.4	57.4	57.4	57.4	57.4	57.4	57.4	57.4	57.4
Mining	%	5.9	5.9	5.9	5.9	5.9	5.9	5.9	5.9	5.9
Services	%	36.7	36.7	36.7	36.7	36.7	36.7	36.7	36.7	36.7

*Total*	*%*	*100*	*100*	*100*	*100*	*100*	*100*	*100*	*100*	*100*

**Table 6:** Electricity consumption in Agriculture, Mining, Services, and urban and rural households from 2018 to 2023.

Sector	Total Electricity Consumption (GWh)					
	2018	2019	2020	2021	2022	2023
Industry	28.06	43.89	38.61	40.28	50	50.56
Agriculture	3.64	5.68	5.01	5.24	6.5	6.57
Mining	24.42	38.21	33.6	35.03	43.5	43.98
Services	97.22	151.67	133.61	140	173.33	175.28
Household (Total)	56.94	88.61	78.06	81.94	101.39	102.5
Household (Urban)	54.09	84.18	74.15	77.85	96.32	97.38
Household (Rural)	2.85	4.43	3.9	4.09	5.07	5.125

*Total*	181.94	284.17	250.56	266.22	325	328.61

**Table 7:** Urban and rural household Electrification rates from 2018 to 2050.

Scenario	Household Electrification Rate (%)									
	2018	2019	2020	2021	2022	2023	2025	2030	2040	2050
Urban	53.2	51.4	55	57	57	57	61.82	73.87	98	100
Rural	6.4	4.7	4.7	4.9	4.9	4.9	10.9	26	56	86

**Table 8:** Historic and predicted sectoral energy intensities per annum from 2018 to 2050.

Scenario	Units	Energy Intensity (MJ/US$)								
		2018	2019	2020	2021	2022	2023	2030	2040	2050
Agriculture	MJ/US$	0.0056	0.0083	0.0074	0.0075	0.0089	0.0088	0.0112	0.0158	0.0222
Mining	MJ/US$	0.3643	0.5415	0.4860	0.4868	0.5813	0.5720	0.7277	1.0265	1.448
Services	MJ/US$	0.2332	0.3456	0.3107	0.3127	0.3723	0.3664	0.3665	0.3665	0.3665
Urban House	MJ/dw/yr	4856.4	7658.5	6032.0	5987.0	7093.1	7026.1	8683.2	11,669.5	15,682.8
Rural House	MJ/dw/yr	2124.7	4408.2	3715.1	3665.5	4342.7	4301.7	4941.3	6023.4	7342.5

**Table 9:** Base, High, and Low GDP growth rate scenarios from 2023 to 2050.

Scenario	GDP Growth Rate (%)							
	2023	2024	2025	2030	2035	2040	2045	2050
Base	2.75	4.74	5.19	4.6	4.6	4.6	4.6	4.6
High	2.75	4.74	5.19	5.19	5.19	5.19	5.19	5.19
Low	2.75	2.75	2.75	2.75	2.75	2.75	2.75	2.75

**Table 10:** Base, High, and Low population growth rate scenarios from 2023 to 2050.

Scenario	Population Growth Rate (%)						
	2023	2025	2030	2035	2040	2045	2050
Base	2.06	2.06	2.06	2.06	2.06	2.06	2.06
High	2.06	2.54	2.54	2.54	2.54	2.54	2.54
Low	2.06	2	1.85	1.7	1.55	1.4	1.25

**Fig. 1:**
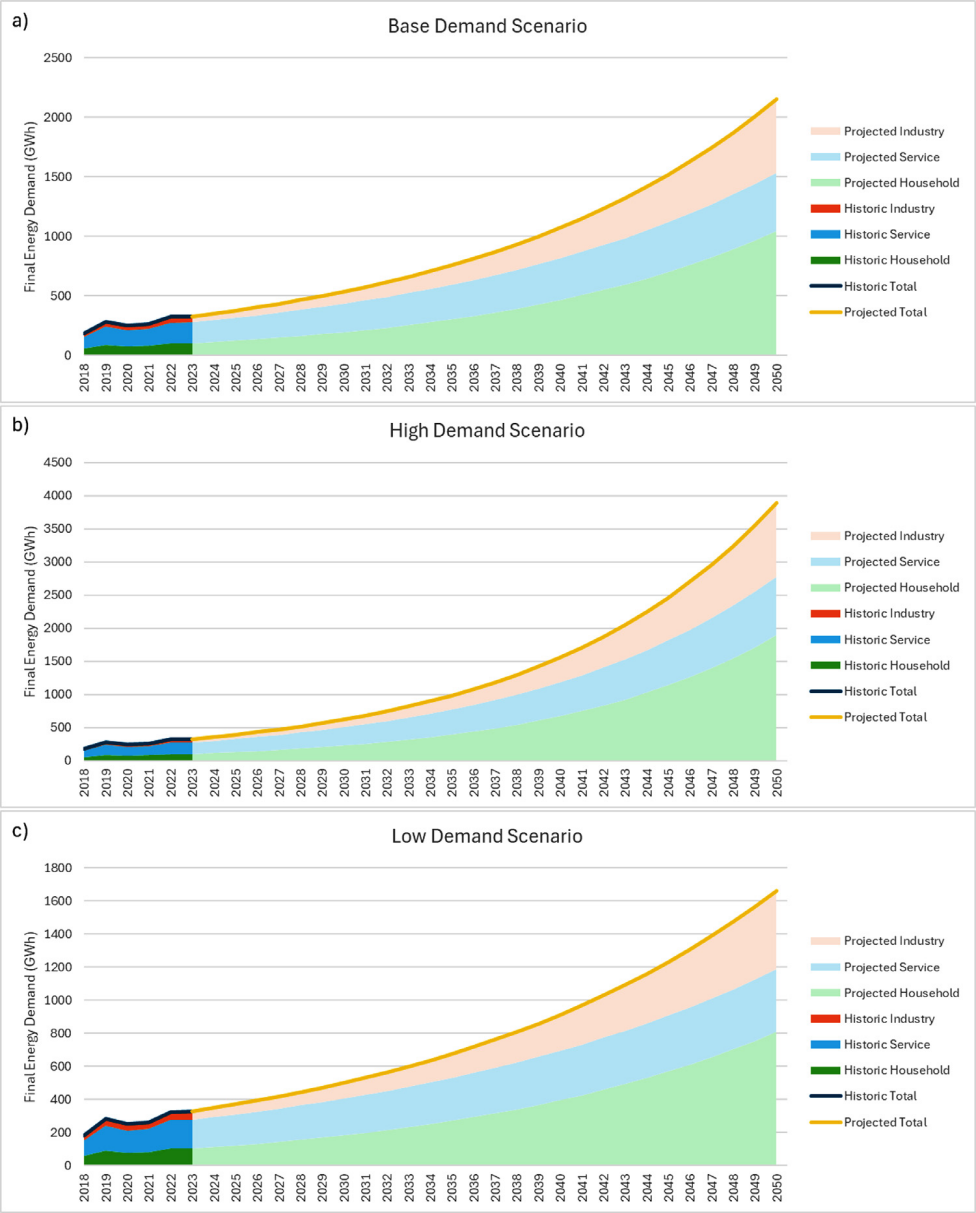
Graphs showing historic and projected electricity demand growth based on IRP data and EDSA historic generation data from Table 1, Table 2. including sectoral breakdown between Household, Service and Industrial sectors. *a) Base demand scenario for the increase in energy demand based on IRP growth rates and EDSA historic generation data. b) High demand scenario based on the high demand IRP scenario growth rates applied to EDSA historic generation data. c) Low demand scenario based on the low demand IRP scenario growth rates applied to EDSA historic generation data.*

**Fig. 2:**
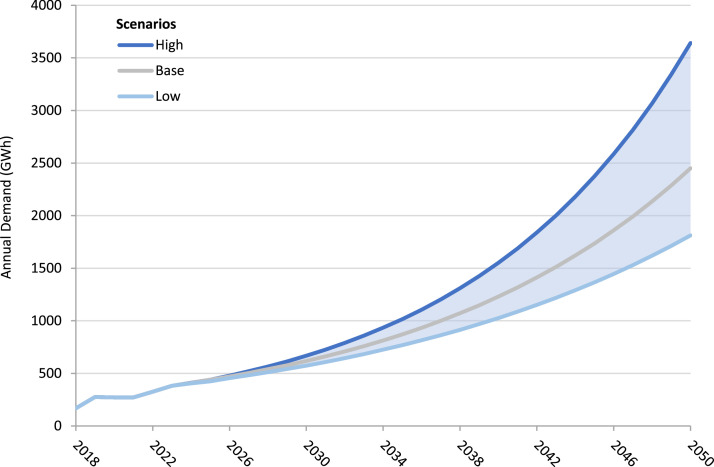
MAED projected final electricity demand under base, high and low scenarios using data from Table 4, Table 5, Table 6, Table 7, Table 8, Table 9, Table 10.

Two illustrative scenarios were developed and included to demonstrate some of the possible development pathways for the growth of Sierra Leone's energy demand. One high demand scenario builds on the baseline to produce ambitious GDP growth alongside higher population growth. The low demand scenario develops the baseline to integrate more reserved GDP growth alongside reduced population growth. This data is summarised in Table 9, Table 10, with the full data set available in the data repository in the Excel File “*Sierra Leone Model Data*” under the sheet labelled “*3. MAED Demand Scenarios*”. The results from these scenarios and the base scenario can also be found under the sheet labelled “*4. Demand Results*” (Figs. 1 and 2).

### 3.2 Capital costs

The data and sources driving the projected capital costs used in the OSeMOSYS modelling for Sierra Leone, summarised by key years in Table 11, while transmission and distribution capital costs can be found in Table 12. Future costs were considered to not vary unless data was available to suggest otherwise. Table 11, Table 12 present a summary of the dataset, the complete version can be found in the data repository in the Excel File “*Sierra Leone Model Data*” in the sheet labelled “*5. Capital Costs*”.

**Table 11:** Projected generation technology capital costs in $/kW from 2023 to 2050.

Technology	Technology Capital Costs ($/kW)			
	2023	2030	2040	2050
Karpowership	0.0001	0.0001	0.0001	0.0001
Bumbuna Hydropower	3000	3000	3000	3000
Betmai Hydropower	2500	2500	2500	2500
Bekongor Hydropower	3000	3000	3000	3000
Singimi Hydropower	2500	2500	2500	2500
Bumbuna I 50 MW	1500	1500	1500	1500
Bumbuna I 88 MW	9375	9375	9375	9375
Bumbuna II 55MW	6818	6818	6818	6818
Hydropower with Reservoir	3000	3000	3000	3000
Large Hydro (>100MW)	3000	3000	3000	3000
Medium Hydro (10–100MW)	2500	2500	2500	2500
Small Hydro (<10MW)	3000	3000	3000	3000
Mini-Grid (Solar) with Storage	3048	2332	1895	1895
Mini-Grid (Diesel)	1086	1086	1086	1086
Mini-Grid (Hydro)	4000	4000	4000	4000
Mini-Grid (Solar Hybrid)	3048	2332	1895	1895
Mini-Grid (Diesel Hybrid)	1086	1086	1086	1086
Solar PV (Utility)	808	740	657	657
Floating Solar PV (Utility)	1169	1169	1169	1169
Electricity Imports (CLSG)	0.0001	0.0001	0.0001	0.0001

**Table 12:** Projected transmission and distribution technology capital costs in USD/kW from 2023 to 2050.

Technology	Technology Capital Costs ($/kW)			
	2023	2030	2040	2050
Transmission (Grid)	365	365	365	365
Distribution (Grid)	2502	2502	2502	2502
Distribution (Mini-Grid)	2502	2502	2502	2502

### 3.3 Operational life

The operational life of each technology represents the standard number of years over which each technology operates following its over-night investment and construction. For technologies that have no capital costs such as Karpowership and electricity imports, this value is set to 1 to reflect the annual nature of the associated tariffs. A summary of this data is found in Table 13, Table 14 below, while a complete list of assumed operational lifetimes for each technology can also be found in the data repository in the Excel File “*Sierra Leone Model Data*” in the sheet labelled “*6. Operational Lifetimes*”.

**Table 13:** Estimated electricity generation technology operational life.

Technology	Operational Life (Years)
Karpowership	1
Bumbuna Hydropower	50
Betmai Hydropower	50
Bekongor Hydropower	50
Singimi Hydropower	50
Bumbuna I 50 MW	50
Bumbuna I 88 MW	50
Bumbuna II 55 MW	50
Hydropower with Reservoir	50
Large Hydro (>100MW)	50
Medium Hydro (10–100MW)	50
Small Hydro (<10MW)	50
Mini-Grid (Solar) with Storage	25
Mini-Grid (Diesel)	25
Mini-Grid (Hydro)	50
Mini-Grid (Solar Hybrid)	25
Mini-Grid (Diesel Hybrid)	25
Solar PV (Utility)	24
Floating Solar PV (Utility)	20
Electricity Imports (CLSG)	1

**Table 14:** Estimated transmission and distribution technology operational life.

Technology	Operational Life (Years)
Transmission (Grid)	70
Distribution (Grid)	70
Distribution (Mini-Grid)	70

### 3.4 Fixed cost projections

The fixed cost associated with the annual cost of maintaining and operating each technology over the period of its lifespan, in proportion to installed capacity, is modelled between 2018 and 2050. Variable future costs for this were used where data was available, otherwise these values were assumed to remain constant over the modelling period. The cost for Karpowership is presented as a range to reflect their variable nature due to tariff negotiations. The fixed costs for each technology used in Sierra Leone's power sector modelling are summarised for key years in Table 15, Table 16 with the complete data-set available through the data repository in the Excel File “*Sierra Leone Model Data*” in the sheet labelled “*7. Fixed Costs*”

**Table 15:** Projected electricity generation technology fixed costs from 2023 to 2050 in USD/kW/year.

Technology	Technology Fixed Costs ($/kW/year)			
	2023	2030	2040	2050
Karpowership	700–836	700–836	700–836	700–836
Bumbuna Hydropower	102	102	102	102
Betmai Hydropower	75	75	75	75
Bekongor Hydropower	90	90	90	90
Singimi Hydropower	75	75	75	75
Bumbuna I 50 MW	45	45	45	45
Bumbuna I 88 MW	281	281	281	281
Bumbuna II 55MW	204	204	204	204
Hydropower with Reservoir	90	90	90	90
Large Hydro (>100MW)	90	90	90	90
Medium Hydro (10–100MW)	75	75	75	75
Small Hydro (<10MW)	90	90	90	90
Mini-Grid (Solar) with Storage	40	30	25	25
Mini-Grid (Diesel)	90	90	90	90
Mini-Grid (Hydro)	120	120	120	120
Mini-Grid (Solar Hybrid)	40	30	25	25
Mini-Grid (Diesel Hybrid)	90	90	90	90
Solar PV (Utility)	10	9	9	9
Floating Solar PV (Utility)	40	40	40	40
Electricity Imports (CLSG)	175	175	175	175

**Table 16:** Projected transmission and distribution technology fixed costs from 2023 to 2050.

Technology	Technology Fixed Costs ($/kW/year)			
	2023	2030	2040	2050
Transmission (Grid)	0	0	0	0
Distribution (Grid)	0	0	0	0
Distribution (Mini-Grid)	0	0	0	0

### 3.5 Variable cost projections

Variable costs are used to model the fuel costs which are scaled by the activity levels of different power-generating technologies. A summary of these values across key years can be found in Table 17. while the complete dataset is available through the data repository in the Excel File “*Sierra Leone Model Data*” in the sheet labelled “*8. Variable Costs*”

**Table 17:** Fuel cost projections in USD/GJ for imported and domestically extracted fuels from 2024 to 2050.

Commodity	Fuel Price ($/GJ)			
	2024	2030	2040	2050
Crude Oil Imports	12.6	14.3	16.9	19.5
Biomass Extraction	1.6	1.6	1.6	1.6
Coal Imports	5.3	5.5	5.9	5.9
LFO (Diesel) Imports	34.3	36.3	39.5	42.6
Heavy Fuel Oil Imports	9.2	10.4	12.3	14.2
Natural Gas Imports	10.5	11.5	13.7	13.7

### 3.6 Emissions factors

To address policy concerns around the emissions associated with each scenario and their respective energy mixes, each fuel used in the model carries an associated emission factor obtained from the Intergovernmental Panel on Climate Change (IPCC). The key data used in OSeMOSYS for these emissions are summarised in Table 18. This data is also available through the data repository in the Excel File “*Sierra Leone Model Data*” under the sheet labelled “*9. Emissions Factors*”.

**Table 18:** Emissions factors for the fuels used in the modelling in kg of CO2e per GJ.

Fuel	CO_2_ Emission Factor (kg/GJ)
Crude Oil	73.3
Biomass	100
Coal	94.6
LFO (Diesel)	74.1
HFO	77.4
Natural Gas	56.1

### 3.7 Efficiencies

Technology-specific efficiencies within the model are used to represent the ratios between energy input from each fuel source, and the energy output from each technology. The data for this was collected from several different sources and can be found in Table 19. as well as being available through the data repository in the Excel File “*Sierra Leone Model Data*” under the sheet labelled “*10. Technology Efficiencies*”. Efficiencies for power generation and transmission technologies were assumed to remain constant throughout the model period while distribution efficiencies were assumed to increase. An outline of transmission and distribution efficiencies can be found in Table 20 while an annual breakdown of these values is found in the Excel file “*Sierra Leone Model Data*” under the sheet labelled “*10. Technology Efficiencies*”.

**Table 19:** Estimated electricity generation technology average efficiencies in%.

Technology	Efficiency (%)
Karpowership	35
Bumbuna Hydropower	100
Betmai Hydropower	100
Bekongor Hydropower	100
Singimi Hydropower	100
Bumbuna I 50 MW	100
Bumbuna I 88 MW	100
Bumbuna II 55MW	100
Hydropower with Reservoir	100
Large Hydro (>100MW)	100
Medium Hydro (10–100MW)	100
Small Hydro (<10MW)	100
Mini-Grid (Solar) with Storage	100
Mini-Grid (Diesel)	35
Mini-Grid (Hydro)	100
Mini-Grid (Solar Hybrid)	100
Mini-Grid (Diesel Hybrid)	35
Solar PV (Utility)	100
Floating Solar PV (Utility)	100
Electricity Imports (CLSG)	100

**Table 20:** Historic and projected efficiencies for transmission and distribution infrastructure from 2023 to 2050.

Technology	Estimated Efficiency of Technology (%)			
	2023	2030	2040	2050
Transmission (Grid)	95*	95	95	95
Distribution (Grid)	50*	53	56	60
Distribution (Mini-Grid)	50	53	56	60

⁎ These values were based on real-world historic data provided by EDSA.

### 3.8 Capacity factors

Capacity factors represent a ratio of energy produced by a technology as a factor of potential energy production assuming continuous operation of each technology throughout the same period. The data for this is summarised for generation technologies in Table 21 and for transmission and distribution in Table 22. This data was collected from several sources, and the complete dataset is available through the data repository in the Excel File “*Sierra Leone Model Data*” in the sheet labelled “*11. Capacity Factors*”.

**Table 21:** Estimated electricity generation technology average capacity factors in%.

Technology	Average Capacity Factor (%)
Karpowership	46
Bumbuna Hydropower (without Yiben)	53
Bumbuna Hydropower (with Yiben)	72
Betmai Hydropower	36
Bekongor Hydropower	36
Singimi Hydropower	36
Bumbuna I 50 MW (without Yiben)	49
Bumbuna I 50 MW (with Yiben)	90
Bumbuna I 88 MW	86
Bumbuna II 55MW	90
Hydropower with Reservoir	36
Large Hydro (>100MW)	36
Medium Hydro (10–100MW)	36
Small Hydro (<10MW)	36
Mini-Grid (Solar) with Storage	15
Mini-Grid (Diesel)	30
Mini-Grid (Hydro)	36
Mini-Grid (Solar Hybrid)	15
Mini-Grid (Diesel Hybrid)	30
Solar PV (Utility)	13
Floating Solar PV (Utility)	11
Electricity Imports (CLSG)	100

**Table 22:** Estimated transmission and distribution technology average capacity factors in Sierra Leone.

Technology	Average Capacity Factor (%)
Transmission (Grid)	100
Distribution (Grid)	100
Distribution (Mini-Grid)	100

Due to the flow of hydro potential from Yiben to Bumbuna, the capacity factors associate with generation at the Bumbuna site varied depending on the addition of the Yiben reservoir, and the scale of development. The capacity factors for each scenario of Bumbuna and Yiben development established through consultation with stakeholders can be found in the excel file “*Sierra Leone Model Data*” in the sheet labelled “*12. Bumbuna Capacity Factors*”.

### 3.9 Residual capacities

The residual capacity for existing and committed generation technologies is outlined in Table 23 while the residual capacities for existing and committed transmission and distribution technologies can be found in Table 24. These technologies are represented as specific projects such as the Bumbuna, Betmai, Bekongor, and Singimi hydropower projects, or grouped into technology classes. The below data represents grid, mini-grid, and stand-alone-systems. The data for this was collected from several sources, and the complete dataset is available through the data repository in the Excel File “*Sierra Leone Model Data*” in the sheet labelled “*13. Residual Capacities*”.

**Table 23:** On and off grid electricity generation technology residual capacities and capacities of committed plants, grouped by technology when relevant, from 2021 to 2050 in MW.

Technology	Residual and Committed Capacity (MW)					
	2021	2022	2023	2030	2040	2050
LFO (Diesel) Power Plant	29.34*	29.34*	31.74*	25.74	19.34	0
HFO Power Plant	33.20*	33.20*	33.20*	58.20	25.00	0
Karpowership	66.00*	66.00*	66.00*	0	0	0
Bumbuna Hydropower (Existing)	50.00*	50.00*	50.00*	50.00	50.00	50.00
Betmai Hydropower	0*	0*	0*	120.00	120.00	120.00
Bekongor Hydropower	0*	0*	0*	28.00	28.00	28.00
Singimi Hydropower	0*	0*	0*	20.00	20.00	20.00
Small Hydro (<10MW)	12.41*	12.41*	12.41*	12.41	12.41	12.41
Mini-Grid (Solar with Storage)	1.33*	1.44*	2.40*	2.40	2.40	0
Mini-Grid (Solar Hybrid)	2.77*	2.77*	2.77*	2.77	2.77	0
Mini-Grid (Diesel Hybrid)	0.74*	0.74*	0.74*	0.74	0.74	0
Solar PV (Utility)	0*	5.00*	5.00*	60.00	60.00	0
Off-Grid Generation (Solar PV)	4.29*	4.29*	0*	0	0	0
Off-Grid Generation (Hydro)	4.89*	4.89*	4.89*	4.89	4.89	0
Electricity Imports (CLSG)	0*	27.00*	27.00*	0	0	0
*Total*	*204.97*	*237.08*	*236.15*	*385.15*	*345.55*	*230.41*

⁎ These values were based historic data for Sierra Leone's power sector capacity from IRENASTAT.

**Table 24:** Residual capacities for transmission and distribution technologies from 2021 to 2050 in MW.

Technology	Residual and Committed Capacity (MW)					
	2021	2022	2023	2030	2040	2050
Transmission (Grid)	70	70	70	70	70	70
Distribution (Grid)	78	78	78	78	78	78
Distribution (Mini-Grid)	3.34	3.59	4.55	4.55	4.55	0

### 3.10 Annual potentials and reserves

Data on the availability of renewable and non-renewable resource potentials is presented in Table 25. More in-depth data on these variables can be found in the data repository under the Excel File “*Sierra Leone Model Data*” in the sheet labelled “*14. Reserves and Potentials”.* Due to a lack of data and lack of domestic potential, wind and geothermal generating technologies were not included in the modelling. No economically recoverable sources of fossil fuels have been discovered within Sierra Leones borders leading to a total reliance on imports to provide fuel to power plants. Hydro storage potential for the Bumbuna and Yiben reservoirs is also summarised in Table 26. This data is available through the data repository under the Excel File “*Sierra Leone Model Data*” in the sheet labelled “*15. Reservoir Energy Potentials*”.

**Table 25:** Fossil fuel and renewable resource potentials for the power sector in Sierra Leone.

Resource	Units	Resource Potential
Solar PV	MW	171,000
Solar CSP	MW	22,500
Large Hydropower(>100MW)	MW	461
Medium Hydropower (10–100MW)	MW	990
Small Hydropower (<10MW)	MW	3000
Wind	MW	0
Biomass	PJ	0
Coal	PJ	0
Natural Gas	PJ	0
Crude Oil	PJ	0
Uranium	PJ	0

**Table 26:** Existing and proposed Bumbuna reservoir energy potentials.

Resource	Units	Resource Potential
Bumbuna Reservoir (Existing)	PJ	0.82
Yiben Reservoir (Planned)	PJ	1.91

### 3.11 Historic generation

Historic generation data for the power sector in Sierra Leone between 2018 and 2023 is summarised in Table 27. This data is aggregated by generation technology for grid connected generation. Mini-grid and stand-alone-systems are not included in this summary. This data can also be found in the data repository under the Excel File “*Sierra Leone Model Data*” in the sheet labelled “*16. Historic Generation*”.

**Table 27:** On and off grid historic power generation in Sierra Leone from 2018 to 2050 in MWh.

Technology	Historic Power Generation (MWh)*					
	2018	2019	2020	2021	2022	2023
LFO (Diesel) Power Plant	13,155	7607	4182	4324	1714	3791
HFO Power Plant	74,865	6647	2904	3764	1714	3791
Karpowership	0	246,712	246,712	246,712	261,177	232,246
Bumbuna I Hydropower	209,488	222,128	222,360	219,073	213,335	228,057
Small Hydro (<10MW)	13,640	14,652	13,813	14,035	2634	2481
Solar PV (Utility)	0	0	0	0	989	7155
CLSG Imports	0	0	0	0	104,034	210,113
Mini-Grid (Solar) with Storage	0	0	0	192	383	383
Mini-Grid (Solar Hybrid)	0	0	0	229	465	465
Mini-Grid (Diesel Hybrid)	0	0	0	100	190	190
*Total*	*311,146*	*497,745*	*489,970*	*488,428*	*586,637*	*688,672*

⁎ These values were based on real-world historic data provided by EDSA and EGTC.

### 3.12 Robust decision-making data

Robust Decision-Making (RDM) is a methodology that tests the robustness of outcomes of a model in relation to changes in chosen variables within pre-defined sets of bounds. RDM uses Latin-Hypercube Sampling (LHS) to vary the selection of variables being tested across multiple runs to assess the sensitivity of results against variations in assessed factors [8]. This assessment can help to inform policy decisions by providing context for the most likely scenarios and how vulnerable each projection is to variations in parameters and outcomes. In the case of Sierra Leone, specific variables were chosen that were perceived to be the most likely to both be susceptible to changes in the outcome of Sierra Leone's power sector development and have a significant impact on the model results. A brief overview of these parameters, the bounds used, and the reason for their selection can be found in Table 28.

**Table 28:** Variable input adjustments and upper and lower bounds for Robust Decision-Making (RDM) modelling.

No.	Description	Variable	Lower Bound	Upper Bound	Link
1	Assesses variations from the base demand scenario within the upper and lower bounds of projected demand	Annual Demand	0.740	1.486	
2	Assesses changes in Yiben and Bumbuna reservoir size on hydro availability across wet and dry seasons due to climate change	Bumbuna and Yiben Activity Upper Limit	0.75	1.25	
3	Assesses the impact of larger-scale development of co-located solar PV with the Bumbuna and Yiben development	Floating Solar Capacity	1.00	4.00	
4	Assesses the impact that changes in the capital costs associated with Solar PV technologies has on capacity mix	Solar PV Capital Costs	0.75	1.25	
5	Assesses the impact that changes in the fixed costs associated with Solar PV technologies has on capacity mix	Solar PV Fixed Costs	0.75	1.25	
6	Assesses the impact that changes in the capital costs associated with Hydropower has on capacity mix	Hydropower Capital Costs	0.75	1.25	
7	Assesses the impact that changes in the fixed costs associated with Hydropower has on capacity mix	Hydropower Fixed Costs	0.75	1.25	
8	Assesses the impacts of capital cost changes associated with Sierra Leone's transmission and distribution infrastructure	T&D Infrastructure Capital Costs	0.75	1.25	
9	Assesses the impact of increased efficiency in Sierra Leone's transmission infrastructure efficiency	Transmission Efficiency	1.00	1.05	
10	Assesses the impact of increased efficiency in Sierra Leone's distribution infrastructure efficiency	Distribution Efficiency	1.00	1.40	
12	Assesses the impact of changes to the efficiency of fossil fuel Plants on Sierra Leone's on energy mix	Fossil Fuel Power Plant Efficiency	0.90	1.00	
13	Assesses the impact of changes in the cost of fossil fuels on Sierra Leones energy mix	Fossil Fuel Costs	0.80	1.50	
14	Assesses the impact of changes in the capital costs associated with fossil fuel power plants on Sierra Leone's energy mix	Fossil Fuel Power Plant Capital Costs	0.75	1.25	
15	Assesses the impact of changes in the fixed costs associated with fossil fuel power plants on Sierra Leone's energy mix	Fossil Fuel Power Plant Fixed Costs	0.75	1.25	
16	Assesses the impact of changes in the costs of Karpowership on Sierra Leone's energy mix	Karpowership Fixed Costs	0.75	1.25	
17	Assesses the impacts of changes in the costs of Sierra Leone's fuel imports	Energy Import Variable Costs	0.75	1.25	
18	Assesses the impact of changes in domestic solar and hydro potential on Sierra Leone's power sector development	Solar and Hydro Resource Potential	0.80	1.20	
19	Assesses the impact of changes in domestic biomass potential on Sierra Leone's power sector development	Biomass Resource Potential	0.80	1.20	
20	Assesses changes in hydro-availability during wet-seasons on Sierra Leones energy mix	Wet-Season Hydro Capacity Factor	0.80	1.20	
21	Assesses changes in hydro-availability due to climate change during dry-seasons on Sierra Leones energy mix	Dry-Season Hydro Capacity Factor	0.80	1.20	

## 4 Experimental Design, Materials and Methods

This dataset was compiled through an extensive literature review of the data available directly related to Sierra Leone's energy sector or for comparable sources in similar regions or power systems. The data was collected from several international databases, academic reports, and national agencies, with the raw data being processed to comply with the data requirements of OSeMOSYS and MAED modelling. The following sections details the methodology, construction, and sources of this database.

### 4.1 Demand projections

To calculate the demand, historic electricity generation data from EDSA [19] found in Table 1 was collected for years between 2018 and 2023. This was then adjusted by transmission and distribution efficiencies as seen in Eq. (1) to generate an estimate of final demand for these years, found in Table 3.

(1) $$\begin{array}{ccc} Final\;Energy\;Demand & = & \left(Generation\;\left[PJ\right]\times \;Transmission\;Efficiency\;\left[\%\right]\right)\\ & & \times \;Distribution\;Efficiency\;\left[\%\right] \end{array}$$

As there was no data available to inform sectoral splits within the “*High*” and “*Low*” scenarios these were assumed to follow the same sectoral splits as the “*Base*” scenario found in the IRP data [19]. As such, sectoral demand was calculated between 2018 and 2023 based on the total annual demand calculated EDSA historic data, divided by sector according to the 2019 sectoral split found in the IRP model as shown in (2), (3) which are summarised in Table 2.

(2) SectoralShare[%]=BaseScenarioSectoralDemand[PJ]/BaseScenarioTotalEnergyDemand[PJ]

(3) SectoralEnergyDemand[PJ]=FinalAnnualEnergyDemand[PJ]×SectoralShare[%]

To model growth in energy demand for future projections, data from previous IRP modelling [19] was used to generate growth rates for “*Base*”, “*High*”, and “*Low*” demand scenarios; to calculate this, the CAGR formula was applied to the change in IRP predictions of energy demand between 2020 and 2040 for each case, as shown in Eq. (4).

(4) $$Demand\;CAGR\;\left[PJ\right]=\left({\left(\frac{2040\;Demand\;\left[PJ\right]}{2020\;Demand\;\left[PJ\right]}\right)}^{1\;/\;20}\right)-1$$

These values were projected forwards using (5), (6).

(5) $$Total\;Demand_{Current\;Year}\;\left[PJ\right]=\;Demand_{2023}\times {\left(1+\;Total\;CAGR\right)}^{Current\;Year-2023}$$

(6) $$SD{\;}_{CY}\;\left[PJ\right]=TD_{CY}\;\left[PJ\right]\times \frac{Sectoral\;Share_{2023}\times {\left(1+\;Sectoral\;CAGR\right)}^{\left(CY-2023\right)}}{\sum All\;Sectoral\;Shares\times {\left(1+\;All\;Sectoral\;CAGR\right)}^{\left(CY-2023\right)}}$$

Where:

SD = Sectoral Demand, CY = Current Year, TD = Total Demand.

The sources for data inputs used in the MAED model are summarised in Table 29, Table 30, Table 31, Table 32. The complete data for this section can be found in the data repository in the Excel File “*Sierra Leone Model Data*” under the sheet labelled “*2. MAED Demand*”.

**Table 29:** List of sources and assumptions for Sierra Leone population demographic parameters (2018–2030).

Variable	Sources	Assumptions
Population	[9,10]	
Population Growth Rate	[11]	Assumed constant from 2028 to 2050
Urban Population	[12]	
Person/Urban Population	[13,14]	Assumed constant over modelling period
Person/Rural Population	[13,14]	Assumed constant over modelling period
Potential Labour Force	[15,16]	Assumed constant over modelling period
Participating Labour Force	[15,16]	Assumed constant over modelling period

**Table 30:** List of sources for Sierra Leone GDP parameters (2018–2028).

Variable	Sources	Assumptions
GDP (US$ Billion)	[17]	
GDP Growth Rate	[10]	Assumed constant from 2028 to 2050
Sectoral Shares of GDP	[18]	
Industry	[18]	Assumed constant over modelling period
Agriculture	[18]	Assumed constant over modelling period
Mining	[18]	Assumed constant over modelling period
Services	[18]	Assumed constant over modelling period

**Table 31:** List of sources for electricity consumption per sector (2018–2023).

Variable	Sources	Assumptions
Industry	[19,20]	
Agriculture	[19,20]	Assumed to be 2 % of industrial energy consumption
Mining	[19,20]	Assumed to be the remaining portion of industrial consumption
Services	[19,20]	
Household (Total)	[19]	
Household (Urban)	[19]	Assumed to be 95 % of total household consumption
Household (Rural)	[19]	Assumed to be 5 % of total household consumption

**Table 32:** List of sources for electrification rates (2018–2050).

Variable	Sources	Assumptions
Urban	[21,22,20]	Annual electrification growth rates projected in line with GoSL Targets
Rural	[21,22,20]	Annual electrification growth rates projected in line with GoSL Targets

Sectoral electricity consumption was calculated assuming agricultural consumption constituted 2 % of industrial energy consumption, with the remaining industrial energy consumption attributed to mining, as outlined in Eqs. (7) and (8).

(7) AgriculturalEnergyConsumption[PJ]=IndustrialEnergyConsumption[PJ]×0.02

(8) $$\begin{array}{ccc} Mining\;Energy\;Consumption\;\left[PJ\right] & = & \;Industrial\;Consumption\;\left[PJ\right]\\ & & -\;Agricultural\;Energy\;Consumption\;\left[PJ\right] \end{array}$$

MAED demand projections were based on the EDSA historic baseline demand between 2018 and 2023, projected using the compound annual growth rates calculated in initial demand modelling, this was then divided between sectors based on their share of GDP using Eq. (9). High and Low demand scenarios were established through modelling changes in both the growth of GDP and of Population. The sources and assumptions for these scenarios can be found in Table 33. and Table 34.

(9) $$Sectoral\;Energy\;Demand\;\left[PJ\right]=Total\;Energy\;Demand\;\left[PJ\right]\;\times \frac{Sectoral\;Share\;of\;GDP\;\left[USD\right]}{Total\;GDP\;\left[USD\right]}$$

**Table 33:** List of sources for GDP growth rate scenarios for Sierra Leone (2023–2050).

Scenario	Sources	Assumptions
Base	[10]	Assumed constant from 2028 to 2050
High	[23]	Assumed to reach 5.19 % from 2025 to 2039 in line with Sierra Leone's Vision 2039 ambitions
Low	[10]	Assumed to remain at 2023 levels until 2050

**Table 34:** List of sources for population growth rate scenarios for Sierra Leone.

Scenario	Sources	Assumptions
Base	[11]	Assumed constant from 2028 to 2050
High	[20]	
Low	[11]	Assumes 0.03 % reduction in line with 2019–2021 rate reductions

### 4.2 Capital costs

Capital cost data was collected from several sources summarised in Table 35. Projected costs were taken from literature, and considered straight between points, except for solar PV technologies who exhibit linear decreases in capital cost. For technologies where cost is dependent on capacity, but not on construction, such as Karpowership, the capital cost associated with these technologies was assumed to be negligible.

**Table 35:** List of sources for technology capital costs.

Technology	Sources	Assumptions
Biomass Power Plant	[24]	
Coal Power Plant	[24]	
LFO (Diesel) Power Plant	[24]	
HFO Power Plant	[24]	
Gas Plant (CCGT)	[24]	
Gas Plant (SCGT)	[24]	
Karpowership	–	Costs set to near 0 for modelling.
Bumbuna Hydropower	[25]	Previous study by MCC.
Betmai Hydropower	[24]	Assumed same as Medium Hydropower Plant
Bekongor Hydropower	[24]	Assumed same as Large Hydropower Plant
Singimi Hydropower	[24]	Assumed same as Medium Hydropower Plant
Bumbuna I 50 MW	[25]	
Bumbuna I 88 MW	[25]	CAPEX for an 88 MW upgrade to the existing Bumbuna site and a new reservoir and 55 MW generator at Yiben is US$ 1.2 Billion
Bumbuna II 55MW	[25]	CAPEX of upgrading existing Bumbuna site by 50 MW and a new reservoir and 55 MW generator at Yiben is US$ 450 Million.
Hydropower with Reservoir	[24]	Assumed same as Large Hydropower Plant
Large Hydro (>100MW)	[24]	
Medium Hydro (10–100MW)	[24]	
Small Hydro (<10MW)	[24]	
Mini-Grid (Solar) with Storage	[26]	
Mini-Grid (Diesel)	[26]	
Mini-Grid (Hydro)	[26]	
Mini-Grid (Solar Hybrid)	[26]	
Mini-Grid (Diesel Hybrid)	[26]	
Solar PV (Utility)	[27]	Linear between data points found in source. Remains at 2040 level when forecast ends
Solar PV (Utility with Storage)	[24,28]	Linear between data points found in source. Remains at 2040 level when forecast ends
Floating Solar PV (Utility)	[29]	Value used lies between the 10 MW and 50 MW values in source.
Off-Grid Generation (Diesel)	[24]	
Off-Grid Generation (Solar PV)	[24]	
Off-Grid Generation (Hydro)	[24]	
Crude Oil Refinery	[24]	
Electricity Imports (Guinea)	[25]	Costs reflected in Fixed Cost parameter to simulate the annual tariffs
Electricity Imports (CLSG)	[25]	Assumed same as CLSG Imports

### 4.3 Operational life

Data for assumed operational lifetimes of power sector technologies for Sierra Leone was collected from several sources. The sources and assumptions made in the collection of this data are summarised in Table 36.

**Table 36:** List of sources for technology lifetimes.

Technology	Sources	Assumptions
Biomass Power Plant	[24]	
Coal Power Plant	[30]	
LFO (Diesel) Power Plant	[30]	
HFO Power Plant	[30]	
Gas Plant (CCGT)	[30]	
Gas Plant (SCGT)	[30]	
Karpowership	–	Set to 1 year to reflect annual nature of tariff.
Bumbuna Hydropower	[24]	Assumed same as Medium Hydropower Plant
Betmai Hydropower	[24]	Assumed same as Medium Hydropower Plant
Bekongor Hydropower	[24]	Assumed same as Large Hydropower Plant
Singimi Hydropower	[24]	Assumed same as Medium Hydropower Plant
Bumbuna I 50 MW	[24]	Assumed same as Medium Hydropower Plant
Bumbuna I 88 MW	[24]	Assumed same as Medium Hydropower Plant
Bumbuna II 55MW	[24]	Assumed same as Medium Hydropower Plant
Hydropower with Reservoir	[24]	Assumed same as Large Hydropower Plant
Large Hydro (>100MW)	[24]	
Medium Hydro (10–100MW)	[24]	
Small Hydro (<10MW)	[24]	
Mini-Grid (Solar) with Storage	[31]	
Mini-Grid (Diesel)	[31]	
Mini-Grid (Hydro)	[24]	Assumed same as Off-Grid Hydropower
Mini-Grid (Solar Hybrid)	[31]	
Mini-Grid (Diesel Hybrid)	[31]	
Solar PV (Utility)	[24]	
Solar PV (Utility with Storage)	[24]	
Floating Solar PV (Utility)	[25]	
Off-Grid Generation (Diesel)	[30]	
Off-Grid Generation (Solar PV)	[24]	Assumed same as On-Grid Solar PV
Off-Grid Generation (Hydro)	[24]	
Crude Oil Refinery	[32]	
Electricity Imports (Guinea)	–	Set to 1 year to reflect annual nature of tariff.
Electricity Imports (CLSG)	–	Set to 1 year to reflect annual nature of tariff.

### 4.4 Fixed cost

Fixed cost data for the operation and maintenance of Sierra Leone's generation capacity was collected from several sources which are summarised in Table 37. Karpowership costs were calculated from reported tariff changes and associated changes in annual costs using Eqs. (10) and (11), with the overall cost using the median of the two values reported. Where data was not available, the fixed costs were assumed to be equal to a percentage of the technologies capital costs as outlined in IRENA's Planning Prospect for Africa (2021) [24].

(10) TariffChange[USD/kWh]=OldTariff[USD/kWh]−NewTariff[USD/kWh]

(11) $$Annual\;Costs\;\left[USD/year\right]=\;Tariff\;\left[USD/kWh\right]\;\times \;\frac{Annual\;Savings\;\left[USD/year\right]}{Tariff\;Change\;\left[USD/kWh\right]}$$

**Table 37:** List of sources for technology fixed costs.

Technology	Sources	Assumptions
Biomass Power Plant	[24]	
Coal Power Plant	[24]	
LFO (Diesel) Power Plant	[24]	
HFO Power Plant	[24]	
Gas Plant (CCGT)	[24]	
Gas Plant (SCGT)	[24]	
Karpowership	[33]	Assumed that Karpowership costs GoSL between $55.18 and $46.18 million USD annually. Calculated from source.
Bumbuna Hydropower	[25,24]	Assumed 3 % of capital cost in line with IRENA methodology
Betmai Hydropower	[24]	Assumed same as Medium Hydropower Plant
Bekongor Hydropower	[24]	Assumed same as Large Hydropower Plant
Singimi Hydropower	[24]	Assumed same as Medium Hydropower Plant
Bumbuna I 50 MW	[24]	Assumed 3 % of capital cost in line with IRENA methodology
Bumbuna I 88 MW	[24]	Assumed 3 % of capital cost in line with IRENA methodology
Bumbuna II 55MW	[24]	Assumed 3 % of capital cost in line with IRENA methodology
Hydropower with Reservoir	[24]	
Large Hydro (>100MW)	[24]	
Medium Hydro (10–100MW)	[24]	
Small Hydro (<10MW)	[24]	
Mini-Grid (Solar) with Storage	[26]	
Mini-Grid (Diesel)	[26]	
Mini-Grid (Hydro)	[26]	
Mini-Grid (Solar Hybrid)	[26]	
Mini-Grid (Diesel Hybrid)	[26]	
Solar PV (Utility)	[23]	
Solar PV (Utility with Storage)	[23]	
Floating Solar PV (Utility)	[25]	
Off-Grid Generation (Diesel)	[24]	
Off-Grid Generation (Solar PV)	[24]	Assumed same as On-Grid Solar PV
Off-Grid Generation (Hydro)	[23]	
Crude Oil Refinery	[34]	TEMBA
Electricity Imports (Guinea)	–	Default for modelling
Electricity Imports (CLSG)	–	Default for modelling

### 4.5 Variable cost projections

Through stakeholder engagement it was established that Sierra Leone lacks commercially viable fossil fuel extraction, and as such domestic fossil fuels were omitted. Variable cost data was for the remaining domestic biomass and imported fossil fuels for generation in Sierra Leone's power sector was collected from several sources which are summarised in Table 38.

**Table 38:** List of sources for technology variable costs.

Resource	Sources	Assumptions
Crude Oil Imports	[30]	
Biomass Extraction	[30]	
Coal Imports	[30]	
LFO (Diesel) Imports	[35]	Equal to Pump Price (30 SLE in 2024). Cost increases in proportion with those seen for diesel in [30]
Heavy Fuel Oil Imports	[30]	
Natural Gas Imports	[24]	

### 4.6 Emissions factors

All emissions factors were calculated based on reports from the 2006 IPCC Guidelines for National Greenhouse Gas Inventories [36], with biomass assumed to align with the category “Other Primary Solid Biomass” in the IPCC report. This was used for CO_2_ emissions, although it does have the scope to include more emissions if required. The data presented in the IPCC report was converted from the reported values in kg/TJ to kg/GJ for use in OSeMOSYS.

### 4.7 Efficiencies

Efficiencies represent the ratio between energy input (input activity ratio) and energy output (output activity ratio). In the started data kits available for OSeMOSYS [37] all technologies besides transmission and distribution have an output activity ratio of 1, and an input activity ratio appropriate to their efficiency, calculated using Eq. (12) and rearranged for the desired variable. Table 39 below summarises the sources used for these efficiencies, and their associated assumptions.

(12) $$Technology\;Efficiency\;\left[\%\right]\;=100\;\times \left(\frac{Output\;Activity\;Ratio}{Input\;Activity\;Ratio}\right)$$

**Table 39:** List of sources for technology efficiencies.

Technology	Sources	Assumptions
Biomass Power Plant	[24]	
Coal Power Plant	[30]	
LFO (Diesel) Power Plant	[30]	
HFO Power Plant	[30]	
Gas Plant (CCGT)	[30]	
Gas Plant (SCGT)	[30]	
Karpowership	[30]	Assumed same as Heavy Fuel Oil Power Plant
Bumbuna Hydropower	[24]	Assumed same as Medium Hydropower Plant
Betmai Hydropower	[24]	Assumed same as Medium Hydropower Plant
Bekongor Hydropower	[24]	Assumed same as Large Hydropower Plant
Singimi Hydropower	[24]	Assumed same as Medium Hydropower Plant
Bumbuna I 50 MW	[24]	Assumed same as Medium Hydropower Plant
Bumbuna I 88 MW	[24]	Assumed same as Medium Hydropower Plant
Bumbuna II 55MW	[24]	Assumed same as Medium Hydropower Plant
Hydropower with Reservoir	[24]	Assumed same as Large Hydropower Plant
Large Hydro (>100MW)	[24]	
Medium Hydro (10–100MW)	[24]	
Small Hydro (<10MW)	[24]	
Mini-Grid (Solar) with Storage	[24]	Default for Modelling
Mini-Grid (Diesel)	[30]	Assumed same as Diesel Power Plant
Mini-Grid (Hydro)	[24]	Default for Modelling
Mini-Grid (Solar Hybrid)	[24]	Default for Modelling
Mini-Grid (Diesel Hybrid)	[30]	Assumed same as Diesel Power Plant
Solar PV (Utility)	[24]	Default for Modelling
Solar PV (Utility with Storage)	[24]	Default for Modelling
Floating Solar PV (Utility)	[24]	Default for Modelling
Off-Grid Generation (Diesel)	[30]	
Off-Grid Generation (Solar PV)	[24]	Default for Modelling
Off-Grid Generation (Hydro)	[24]	Assumed same as Small Hydropower Plant
Crude Oil Refinery	[38]	
Electricity Imports (Guinea)	–	
Electricity Imports (CLSG)	–	
Transmission	[19]	From stakeholder engagement with EDSA
Distribution	[19]	From stakeholder engagement with EDSA

### 4.8 Capacity factors

Technology capacity factors were collected from several sources summarised in Table 40. This capacity factor is calculated from the real annual generation for each technology, as a factor of the theoretical maximum generation as outlined in Eq. (13). Where data was not complete, Table 40. summarises key assumptions made.

(13) $$Annual\;Capacity\;Factor\;=\;\frac{Annual\;Power\;Generation\;\left[GWh\right]}{Theoretical\;Max\;Power\;Generation\;\left[GWh\right]}$$

**Table 40:** List of sources for average technology capacity factors.

Technology	Sources	Assumptions
Biomass Power Plant	[39]	
Coal Power Plant	[39]	
LFO (Diesel) Power Plant	[39]	
HFO Power Plant	[39]	
Gas Plant (CCGT)	[39]	
Gas Plant (SCGT)	[39]	
Karpowership	[19]	Calculated from monthly generation statistics provided by EDSA.
Bumbuna Hydropower	[25,40]	Assumed that Bumbuna operates at 90 % efficiency in wet-seasons, and 16 % efficiency during dry-seasons based on data on Bumbuna
Betmai Hydropower	[41]	Assumed same as Medium Hydropower Plant
Bekongor Hydropower	[41]	Assumed same as Large Hydropower Plant
Singimi Hydropower	[41]	Assumed same as Medium Hydropower Plant
Bumbuna I 50 MW	[25,40]	
Bumbuna I 88 MW	[25,40]	
Bumbuna II 55MW	[25,40]	
Hydropower with Reservoir	[41]	Assumed same as Large Hydropower Plant
Large Hydro (>100MW)	[41]	
Medium Hydro (10–100MW)	[41]	
Small Hydro (<10MW)	[41]	
Mini-Grid (Solar) with Storage	[42]	Assumed same as On-Grid Solar PV with Storage
Mini-Grid (Diesel)	[39]	Assumed same as LFO (Diesel) Power Plant
Mini-Grid (Hydro)	[41]	Assumed same as Small Hydropower Plant
Mini-Grid (Solar Hybrid)	[42]	Assumed same as On-Grid Soar PV
Mini-Grid (Diesel Hybrid)	[39]	Assumed same as LFO (Diesel) Power Plant
Solar PV (Utility)	[42]	
Solar PV (Utility with Storage)	[42]	Assumed same as On-Grid Solar PV, but capacity factor manually extended by 2 h into night in each season
Floating Solar PV (Utility)	[43]	Assumed to lie between On-Grid Solar PV and lower bound reported in the stated source.
Off-Grid Generation (Diesel)	[30]	
Off-Grid Generation (Solar PV)	[42]	Assumed same as On-Grid Solar PV with Storage
Off-Grid Generation (Hydro)	[41]	
Crude Oil Refinery	–	Default for Modelling
Electricity Imports (Guinea)	–	Default for Modelling
Electricity Imports (CLSG)	–	Default for Modelling

Capacity factors for the existing Bumbuna hydropower plant and the associated upgrades when an additional reservoir at Yiben is not constructed were calculated using Eq. (14) to Eq. (16). The potential energy contained in the Bumbuna reservoir at any given moment was calculated using Eq. (14). The capacity at which the generators can operate throughout the dry season was thus calculated using Eq. (15). The resulting capacity factors were calculated using the capacity of installed generators and Eq. (16).

(14) $$\begin{array}{ccc} Potential\;Energy\;\left[J\right] & = & Density\;of\;Water\;\left[\frac{kg}{m^{3}}\right]\times Volume\;\left[m^{3}\right]\times Gravity\;Acceleration\left[\frac{m}{s^{2}}\right]\\ & & \times Head\;Height\;\left[m\right] \end{array}$$

(15) TotalMaxCapacity[MW]=HoursinHalfaYear[h]×PotentialEnergyStoredinReservoir[MWh]

(16) $$Capacity\;Factor=\frac{Total\;Max\;Capacity\;\left[MW\right]}{Capacity\;of\;Generators\;\left[MW\right]}$$

Capacity factors for the existing Bumbuna hydropower plant and the associated upgrades when an additional reservoir at Yiben is constructed were calculated using Eq. (17) and (18). The potential energy contained in the Yiben reservoir at any given moment was assumed to be a factor of 10 larger than that stored in the existing Bumbuna reservoir; this was ascertained through stakeholder engagement [25]. The total capacity at which the generators can operate throughout the dry season was thus calculated using Eq. (15). This total was used to limit the Yiben generator which was then split by the downstream generators using Eqs. (17) and (18). The resulting capacity factors were calculated using the capacity of installed generators and Eq. (19).

(17) $$\begin{array}{ccc} Max\;Capacity_{Generator\;1}\;\left[MW\right] & = & Total\;Max\;Capacity\;\left[MW\right]-(Total\;Max\;Capacity\;\left[MW\right]\\ & & -\;Capacity\;of\;Generator\;1\left[MW\right]) \end{array}$$

(18) $$Max\;Capcity_{Generator\;2}\;\left[MW\right]=Total\;Max\;Capacity\left[MW\right]-Max\;Capacity_{Generator\;1}\;\left[MW\right]$$

(19) $$Capacity\;Factor_{Generator\;1\;or\;2}=\frac{Max\;Capacity_{Generator\;1\;or\;2}\left[MW\right]}{Capacity\;of\;Generator_{Generator\;1\;or\;2}\left[MW\right]}$$

### 4.9 Residual capacities

Residual installed capacity for on-grid technologies was calculated for data available through the IRENASTAT online data query tool [44]. Capacities beyond 2023 were calculated based on the lifespan of the project from its first year of operation, as well as committed projects such as the Bekongor and Betmai hydropower projects.

Residual capacities for mini-grid technologies were established through consultation with the ministry of energy and the Sierra Leone Electricity and Water Regulatory Commission (SLEWRC) [45].

Residual capacities for on-grid transmission and distribution, and mini-grid distribution infrastructure was obtained from consultations with the Sierra Leone Ministry of Energy [25] and EDSA [19].

### 4.10 Annual potential and reserves

Data for the resource potential in Sierra Leone was collected from several sources. Wind was excluded from the domestic reserves as consultation with the Ministry of Energy's Planning Unit indicated no plans to develop wind capacity. Similarly, the Ministry of Energy confirmed that at present there are no commercially viable fossil fuel reserves. Biomass was also excluded from reserves due to a lack of biomass availability for power plants [46]. The sources and assumptions for these annual potentials and reserves are summarised below in Table 41.

**Table 41:** List of sources for annual potential and reserves in Sierra Leone.

Resource	Source	Assumptions
Solar PV	[47]	
Solar CSP	[47]	
Large Hydropower(>100MW)	[25]	
Medium Hydropower (10–100MW)	[25]	
Small Hydropower (<10MW)	[25]	
Wind	[25]	Omitted following consultation with Ministry of Energy
Biomass	[25]	Omitted following consultation with Ministry of Energy
Coal	[25]	Omitted following consultation with Ministry of Energy
Natural Gas	[25]	Omitted following consultation with Ministry of Energy
Crude Oil	[25]	Omitted following consultation with Ministry of Energy
Uranium	[25]	Omitted following consultation with Ministry of Energy

To estimate the annual energy potential of the Bumbuna and Yiben reservoirs, the potential energy calculated using Eq. (14) is added to energy generated during the wet season, as shown by Eq. (20). In the case of the existing Bumbuna reservoir, this equates to the amount of energy a 50 MW turbine can produce while operating at 90 % capacity throughout the wet season (six months). In the case of the planned Yiben reservoir, it equates to the amount of energy a 55 MW turbine can produce while operating at 90 % capacity throughout the wet season.

(20) $$\begin{array}{ccc} Annual\;Potential\;Energy\;\left[MWh\right] & = & Potential\;Energy\;\left[MWh\right]+(Capacity\;of\;Generator\;\left[MW\right]\\ & & \times \;0.9\;\times Hours\;in\;Half\;a\;Year\;\left[h\right]) \end{array}$$

### 4.11 Historic generation

Historic power generation is calculated from annual reports provided by the Electricity Generation and Transmission Company (EGTC) and EDSA. Total generation was disaggregated into generic generation technologies and specific projects. For missing data-points, assumptions regarding annual averages are outlined in Table 42. below.

**Table 42:** List of sources for historic generation data in Sierra Leone (2018–2023).

Technology	Source	Assumptions
LFO (Diesel) Power Plant	[19,48]	2021 value assumed to equal an average of known years
HFO Power Plant	[19,48]	2021 value assumed to equal an average of known years
Karpowership	[19,48]	2019, 2021 values assumed to equal an average of 2022 and 2023 values
Bumbuna I Hydropower	[19,48]	2021 value assumed to equal an average of known years
Small Hydro (<10MW)	[19,48]	2021 value assumed to equal an average of known years
Solar PV (Utility)	[19,48]	
CLSG Imports	[19,48]	
Mini-Grid (Solar) with Storage	[19,48]	2023 Mini-Grid generation assumed to equal 2022 generation
Mini-Grid (Solar Hybrid)	[19,48]	2023 Mini-Grid generation assumed to equal 2022 generation
Mini-Grid (Diesel Hybrid)	[19,48]	2023 Mini-Grid generation assumed to equal 2022 generation

### 4.12 Robust decision-making bounds

To assess the impact that changes in key parameters within the model impacts the overall predicted capacity demands and energy balance within Sierra Leone, Robust Decision-Making bounds are established, and the model is assessed for variation within these bounds. To calculate the bounds for Demand, the “*High*” and “*Low*” case demands for 2050 were used as factors of the “Base” case, as shown in Eqs. (21) and (22).

(21) $$Demand\;Upper\;Bound\;Factor\;=\;\frac{2050\;High\;Demand\;Scenario\;Demand\;\left[PJ\right]\;}{2050\;Base\;Demand\;Senario\;Demand\;\left[PJ\right]\;}$$

(22) $$Demand\;Lower\;Bound\;Factor\;=\;\frac{2050\;Low\;Demand\;Scenario\;Demand\;\left[PJ\right]\;}{2050\;Base\;Demand\;Senario\;Demand\;\left[PJ\right]}$$

Distribution and Transmission Efficiencies were tested to assess the impacts of increasing the infrastructural efficiency, as such these were tested with an upper-bound defined by the scope for improvement possible based on their initial efficiencies in the Base scenario as shown in Eq. (23).

(23) $$Efficiency\;Upper\;Bound\;=1\;+\;\left(\frac{100\;-\;Base\;Case\;Efficiency\;\left(\%\right)\;}{\;Base\;Case\;Efficiency\;\left(\%\right)}\right)$$

Co-located floating solar capacity associated with the Bumbuna expansion plans was set to 25MW in the base scenario. However, consultation with stakeholders suggested that there would be some interest in the effects of increasing this capacity to 100MW. As such the annual minimum capacity and annual max capacity for floating solar capacity PWRSOL005 was tested in RDM using a lower bound of 1 as the base case, and an upper bound calculated using the 100MW as a factor of the base case 25MW of solar capacity (upper bound = 4), as seen in Eq. (24)

(24) $$Floating\;Solar\;Capacity\;Upper\;Bound\;=\;\frac{Upper\;Floating\;Solar\;Capacity\;\left(MW\right)\;}{Base\;Floating\;Solar\;Capacity\;\left(MW\right)}$$

When assessing variables with a likely skew in their potential variations from the base case, such as fossil fuel variable costs and efficiencies, these skews were accounted for in the ranges outlined in RDM. In the case of fossil fuel prices, these are likely to become more expensive with time and as such, the bounds were skewed towards higher variations in the Variable Costs of fossil fuels in OSeMOSYS.

Where there was no available data to base the bounds for each variable assessed, standard values of ± 0.25 factors (High bound = 1.25, Low bound = 0.75) were used unless context suggested that there may be some degree of skew in the variation, such as may be seen in fuel costs.

## Limitations

Some of the data described here is derived from international databases and so not specific to Sierra Leone. Where Sierra Leone-specific data could be found, either online or through stakeholder consultation, it was included. However, there is a significant lack of suitable available data from national institutions.

## Ethics Statement

The authors have ensured that this paper meets the ethics criteria required for publication with regards to social and environmental welfare and the fair use of data. The work does not involve studies with animals or humans.

## CRediT authorship contribution statement

**Fynn Kiley:** Conceptualization, Methodology, Formal analysis, Investigation, Data curation, Writing – review & editing. **David Caulker:** Conceptualization, Methodology, Investigation, Data curation. **William Collier:** Conceptualization, Methodology, Formal analysis, Investigation, Data curation, Writing – review & editing. **Neve Fields:** Conceptualization, Methodology, Formal analysis, Investigation, Data curation, Writing – review & editing. **William Blyth:** Supervision, Conceptualization, Methodology. **Jairo Quirós-Tortós:** Supervision, Conceptualization, Methodology, Writing – review & editing. **Mark Howells:** Supervision, Conceptualization, Writing – review & editing.

## Data Availability

Socio- and Techno-Economic Dataset for Energy Modelling in Sierra Leone (Original data) (Zenodo)
